# A novel approach in diagnosing multiple dentigerous cysts using CBCT illustration indicative of Mucopolysaccharidosis VI – a case report

**DOI:** 10.25122/jml-2021-0288

**Published:** 2022-04

**Authors:** Shalu Rai, Deepankar Misra, Akansha Misra, Ankit Jain, Ashish Verma, Dimple Grover, Ayesha Haris

**Affiliations:** 1. Department of Oral Medicine and Radiology, Institute of Dental Studies and Technologies, Kadrabad, India; 2. Department of Oral Pathology, Institute of Dental Studies and Technologies, Kadrabad, India; 3. Department of Oral Medicine and Radiology, DJ Dental College and Research Centre, Modinagar, India; 4. Department of Periodontology, Sudha Rastogi College of Dental Sciences and Research, Faridabad, India; 5. Department of Oral and Maxillofacial Surgery, Sudha Rastogi College of Dental Sciences and Research, Faridabad, India; 6. Project Officer, Institute of Liver and Biliary Sciences, Delhi, India

**Keywords:** Mucopolysaccharidoses VI diagnosis, corneal opacity, N-Acetylgalactosamine-4-Sulfatase, glycosaminoglycans

## Abstract

Mucopolysaccharidosis VI is a genetic disorder affecting multiple organs with sundry clinical presentations. The main etiological factor reflects the disturbances in mucopolysaccharide metabolism leading to deposition of acid mucopolysaccharide in various tissues. The pathognomonic features of the disease include a large head, short neck, corneal opacity, open mouth associated with an enlarged tongue, enlargement of the skull, and long anteroposterior dimension with unerupted dentition, dentigerous cyst-like follicles, condylar defects, and gingival hyperplasia. An 18-year-old boy with Maroteaux-Lamy syndrome (mucopolysaccharidosis type VI) is described in this article, emphasizing the oral manifestations and radiographic illustration of lesions in the jaws. It also emphasizes the essential role of cone-beam computed tomography to identify and analyze multicentric pathologies in the jaws.

## Introduction

Mucopolysaccharidosis: MPS VI (Maroteaux-Lamy syndrome) is a rare genetic disorder caused by glycosaminoglycans (GAG) storage enzyme deficiency: N-acetylgalactosamine sulfatase. Normally this enzyme is found in the skin, tendons, blood vessels, airways, and heart valves [1, 2]. The etiology of the disease is attributed to the arylsulfatase B gene (ARSB) mutation, which further leads to a deficiency of N-acetylgalactosamine sulfatase. This lysosomal enzyme is essential for the catabolism of GAG, dermatan sulfate (DS), and chondroitin sulfate (CS) [3].

ARSB mutation culminates in excessive accumulation of GAG and DS in the cells, which triggers the tumor necrosis factor (TNF) pathway [4]. This elicits an inflammatory response leading to chondrocyte apoptosis, causing progressive arthropathy. Therefore, these patients usually show significant symptoms involving bone, cartilage, liver, spleen, ligaments, joints, heart valves, airways, meninges, and corneas. The heterogeneity of the clinical presentation is mainly due to genotype-phenotype correlation in ARSB mutations according to residual enzyme activity [5, 6]. Symptoms include growth stunting, coarse facies, thick hair, skeletal deformities, frequent upper airway infections, hepatosplenomegaly, hearing loss, sleep apnea, and stiff joints [2].

Elevated GAGs in the urine and high DS concentrations are markers of disease activity but alone, these are not diagnostic methods. The criterion for the diagnosis is based on the estimation of ARSB enzyme activity confirmed in cultured fibroblasts or isolated leukocytes (value <10% the lower limit of normal). It is estimated that the urinary score of GAG is directly proportional to the progression of the disease [7]. 

Though various treatments have been mentioned in the literature, an interdisciplinary approach is a prerequisite for the management of patients with MPS VI [8, 9].

The objective of this article was to report the case of an 18-year-old male patient in Western Uttar Pradesh, India, seeking endodontic treatment for painful swelling of maxillary anterior teeth. He was diagnosed with MPS VI, focusing on the general characteristics of MPS VI and radiological findings. It also emphasizes the importance of radiographic examination of all unerupted teeth, using Cone Beam Computed Tomography (CBCT) to better delineate the extent of the lesion and its relationship to adjacent anatomical structures.

## Case Report

An 18-year-old male patient reported to the Department of Oral Medicine and Radiology, IDST Modinagar, seeking treatment for orofacial swelling and purulent discharge in left upper front teeth for 6 months. The personal history revealed that both his parents were middle-aged in the fourth decade of life. 

**Figure 1.**
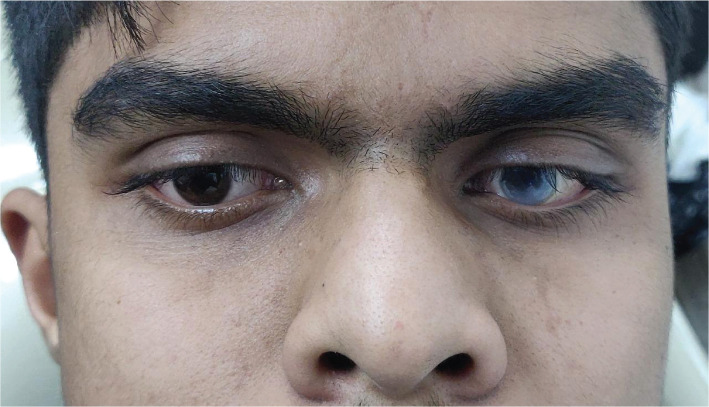
Clinical photograph showing corneal opacity.

**Figure 2.**
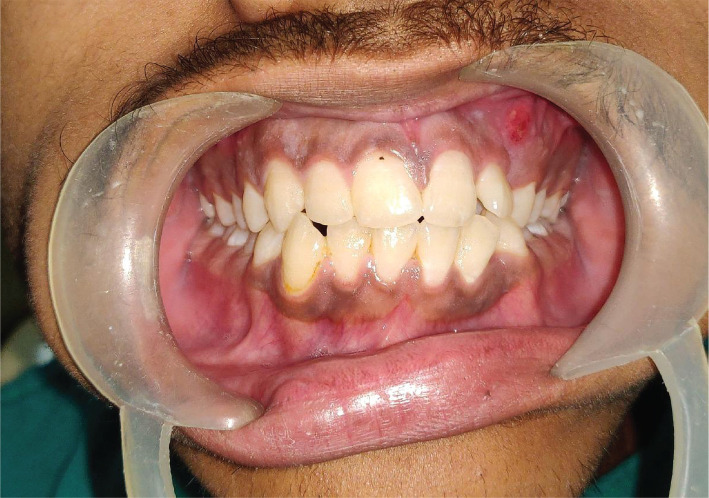
Clinical photograph showing deranged occlusion and gum boil in respect to left maxillary lateral incisor.

His mental status was normal; however, cardiac and broncho-pulmonary insufficiency and mild hepato-splenomegaly were noted. The patient had a large head with corneal clouding in the left eye, flattened nasal bridge, bushy eyebrows, and hypertelorism (Figure 1). Intraoral soft tissue examination revealed macroglossia, thick lips, and distended adenoids, whereas hard tissue examination revealed delayed tooth eruption and a high arched palate. Vestibular obliteration and tenderness were noted in relation to the left maxillary canine (23) along with the presence of sinus tract. An ill-defined swelling was noted in the same region, insidious in onset and relieved on its own with pus discharge (Figure 2). The swelling was soft in consistency and non-compressible, non-fluctuant, and non-reducible. The patient was in good health and reported no history of local trauma, chronic inflammation, cranial nerve alterations, or hearing changes.

**Figure 3.**
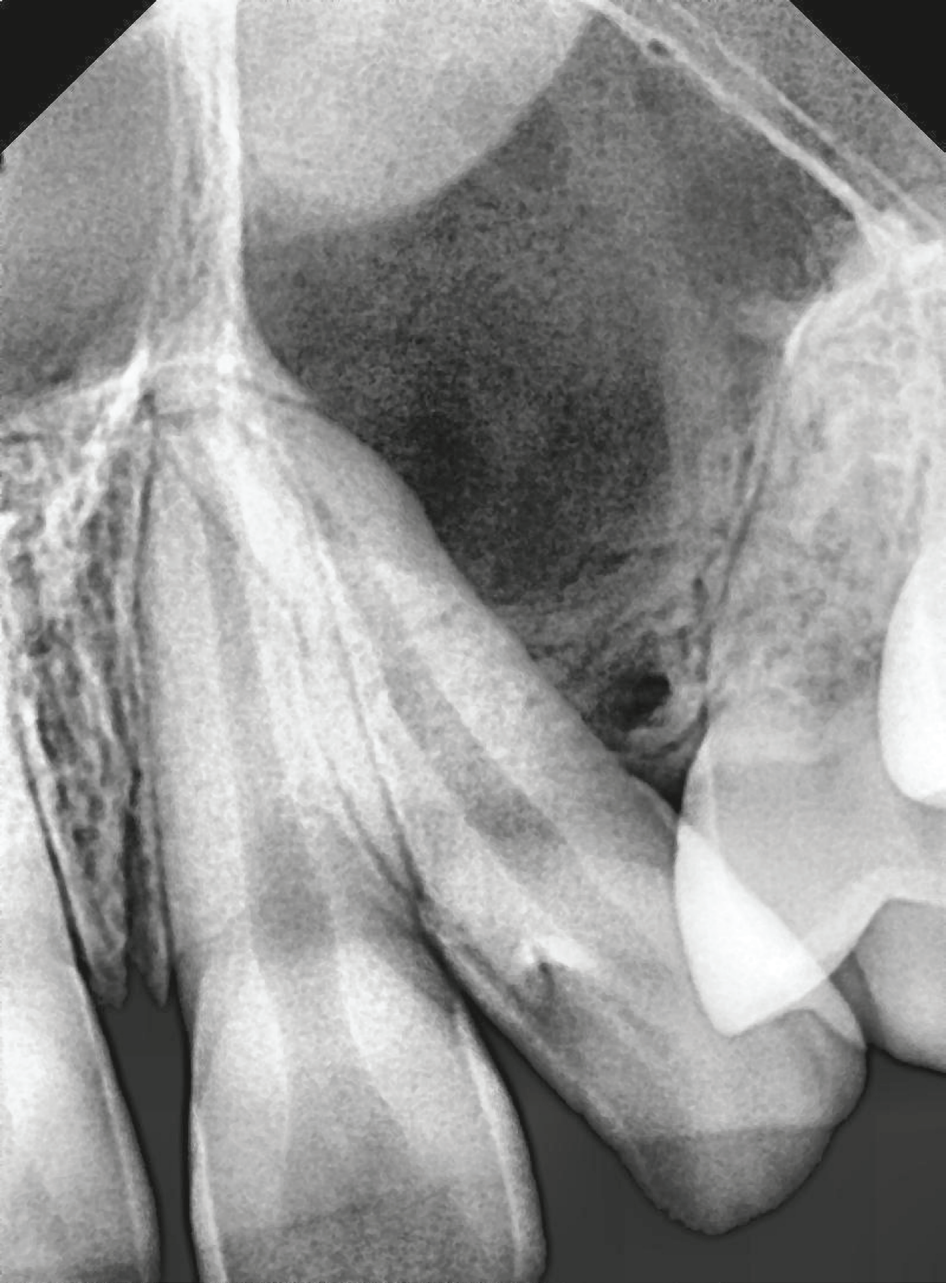
Ill defined radiolucency between roots of maxillary lateral incisor and first premolar causing deflected roots of the teeth and partial image of radiopacity superior to the radiolucency.

**Figure 4.**
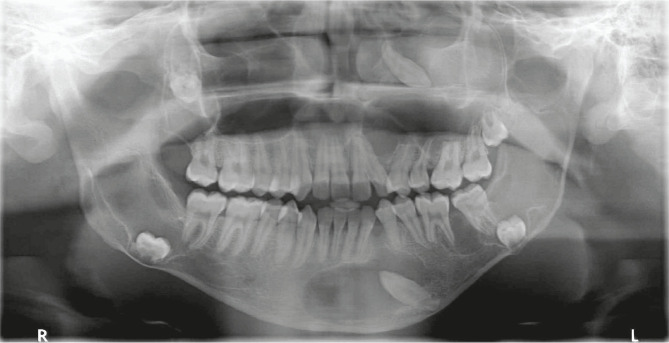
Panoramic radiograph showing multiple cyst-like lesions associated with unerupted teeth in the jaws.

The patient had a radiological examination of the local lesion concerning 23, and intraoral periapical (IOPA) revealed two radiolucencies. A smaller, well-defined radiolucency at the alveolar crest measuring approximately 3 mm × 2.5 mm in greatest diameter between the left lateral incisor and first premolar. Larger radiolucency was associated with a partial image of radiopacity, causing deflection of roots of the left lateral incisor and first premolar suggestive of cystic lesion associated with an unerupted impacted tooth (Figure 3). Therefore, to demarcate the extent of the cystic lesion, the patient was subjected to a panoramic radiograph, which revealed multiple unerupted teeth with pericoronal radiolucencies resembling dentigerous cyst in the mandible and maxilla (Figure 4).

CBCT was acquired to assess the details of lesions manifested in both jaws. The tomographic images were obtained with a CS 9300 scanner at 0.18 mm × 0.18 mm × 0.18 mm resolution (Carestream Health, Inc, Rochester NY, USA) using 90 kV, 15 mA, and 12 seconds of exposure time. The exposure settings were automatically optimized for the patient's size. The voxel size was 300 micrometers. The images were evaluated by an experienced oral and maxillofacial radiologist using CS 3D imaging software (version 3.2.9.0-B, Kodak Dental Systems, Carestream, Rochester, New York, United States) in a dimly lit room on a 17 inch Dell monitor (Intel R Xenon R). 

**Figure 5.**
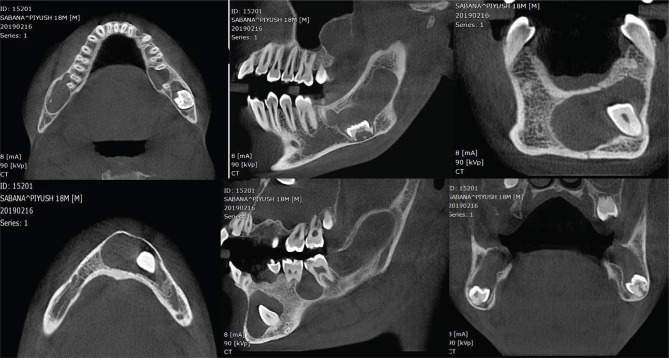
Axial, sagittal and coronal sections showing multiple dentigerous cyst in the mandible.

**Figure 6.**
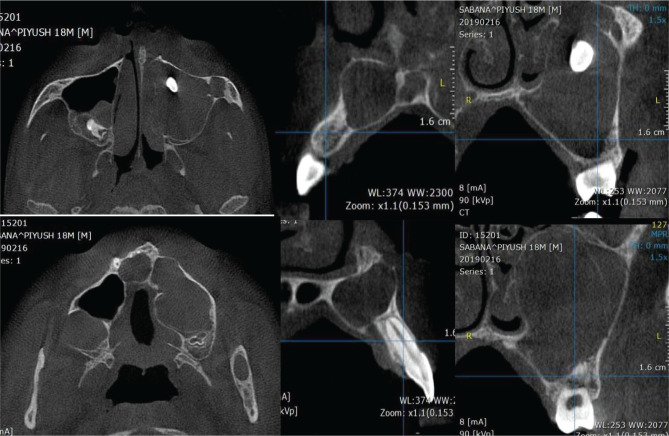
Axial, sagittal and coronal sections showing multiple dentigerous cysts in the maxilla showing severe expansion of the bone.

**Figure 7.**
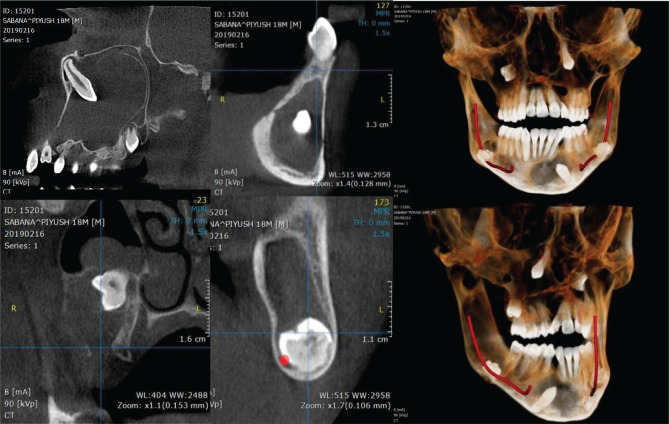
Cross sections of maxilla and mandible with 3-Dimensional reconstruction of jaws depicting multiple dentigerous cyst in the jaws.

CBCT images showed multiple well-defined expansile lesions described below (Figures 5, 6 and 7). First lesion measuring 1.9 cm × 2.4 cm × 2.2 cm, in the greatest antero-posterior (AP), transverse (T) and supero-inferior (SI) dimensions, respectively, in the right posterior maxillary alveolus corresponding to the 18 region and posterior third of the right maxillary sinus. The developing tooth 18 was displaced cranially into the posterior middle third of the right maxillary sinus. The lesion involved the distal peri-radicular region of tooth 17 and the periapical region of the distobuccal and palatal roots of 17. The margins were distinct and thin corticated and appeared attached to the cement-enamel junction (CEJ) of the tooth 18. The internal structure was completely radiolucent with no internal calcifications/septation. Effacement was seen of the adjacent crest and buccal cortex of the alveolus. Thinning, superior displacement, and partial effacement was seen on the adjoining floor of the right maxillary sinus. Blunting suggestive of apical root resorption was seen on the distobuccal and palatal root apices.

Second lesion measuring 5.5 cm × 3.6 cm × 4.3 cm in AP-T-SI dimensions, respectively, in the left maxilla, including the left hard palate and the left maxillary sinus extending anteriorly across the midline to the 12 region. The lesion involved the periapical region of the teeth 12 until 28 and occupied the entire left maxillary sinus cavity. Tooth 23 was seen within the lesion in the anterior superior third of the left maxillary sinus. The margins were well defined, thin corticated, and scalloped. Partial septations were also noted in the inferior third of the lesion. The internal structure was completely radiolucent with no internal calcifications. Apical root resorption was seen in teeth 26, 25, 11, and 12. The lesion involved the nasopalatine canal with effacement of the cortical outlines. Thinning and mild expansion of the labio-buccal cortex was present. In addition, thinning and expansion of the adjacent palatal cortex were noted.

Third lesion measuring 3.14 cm × 1.2 cm × 3.4 cm in AP-T-SI dimensions, respectively, in the right posterior mandible and right mandibular ramus extending from the 47 region till the posterior third of the right mandibular ramus. Supero-inferiorly the lesion involved the entire occluso-apical extent of the alveolus in the 48 region. It involved the buccal peri-radicular region of tooth 47. Thin corticated scalloped margins were seen. Internal contents were completely radiolucent with no internal calcifications/septations. The developing tooth 48 was displaced inferiorly, and the lesion appeared attached to the CEJ of the tooth. Effacement was seen on the crest of the alveolus in the 48 region. There was a thinning and mild expansion of the adjacent buccal and lingual cortices. There was a medial and inferior displacement of the adjacent right inferior alveolar canal. The vertical segment of the canal was displaced posteriorly.

Fourth lesion measuring 1.2 cm × 3.1 cm × 3.0 cm in AP-T-SI dimensions respectively, in the anterior and left parasymphysis region, extending from the 34 region across the midline till the 43 region. The lesion involved the pericoronal region of the mesioangularly impacted tooth 33. The lesion also involved the apical alveolar-basal bone and the periapical region of teeth 32, 34. It extended into the interdental alveolar bone between teeth 32–34 with effacement of the alveolus crest. Thin corticated scalloped margins were observed. Internal contents were completely radiolucent with no internal calcifications/septations. There was a thinning and mild expansion of the adjacent labio-buccal cortex. Mild thinning of the lingual cortex and inferior cortex was observed. Apical root resorption was seen in teeth 34, 32. 

Fifth lesion measuring 1.5 cm × 1.1 cm × 1.4 cm in AP-T-SI dimensions respectively, involving the interdental alveolar bone between teeth 36–37. The lesion involved the distal-lingual peri-radicular region of the distal root of 36 and the mesial-lingual peri-radicular region of the mesial root 37. The margins were thin corticated with completely radiolucent internal contents. There was a thinning and mild expansion of the adjacent lingual cortex. Effacement was seen along the crest of the alveolus lingual to the roots of teeth 36–37. The lesion was caudal to the left inferior alveolar canal.

Sixth lesion measuring 3.5 cm × 1.1 cm × 4.6 cm in AP-T-SI dimensions respectively, in the left posterior mandible and left mandibular ramus extending from the 37 region till the posterior border of the left mandibular ramus. Supero-inferiorly the lesion involved the entire occluso-apical extent of the alveolus-basal bone and the craniocaudal extent of the ramus. It involved the buccal and distal peri-radicular region of tooth 37. The developing tooth 38 was displaced inferiorly, and the lesion appeared attached to the CEJ of the tooth. Thin corticated scalloped margins were observed. Internal contents were completely radiolucent with no internal calcifications/septations. Effacement was seen on the crest of the alveolus in the 38 region. There was thinning of the adjacent buccal and lingual (medial and lateral) cortices and anterior cortex of the ramus. A mild expansion was seen of the medial cortex of the ramus. There was a medial and inferior displacement of the adjacent left inferior alveolar canal. The vertical segment of the canal was displaced posteriorly.

Clinical and radiographic findings indicated a syndrome such as cleidocranial dysplasia, basal cell nevus syndrome, Klippel-Feil syndrome, Maroteaux-Lamy syndrome suffering from multiple dentigerous cysts. The patient was advised to take a posteroanterior chest x-ray and anteroposterior skull and neck radiograph to detect osseous abnormalities in these syndromes, but the radiographs appeared normal. 

On laboratory investigations, thyroid profile, serum calcium, phosphorus levels, and 25-hydroxy cholecalciferol levels were normal. Metabolic screening tests for GAGs in the urine and aryl sulfate B assay were advised to determine Maroteaux-Lamy syndrome. GAG concentration in the urine was 134 mg GAG/g creatinine (normal range 19.97–110.53). Enzyme assay revealed a deficiency of aryl sulfate B that was 83.26nmol/mg proton/h (normal value >121 nmol/mg proton/h). The deranged values indicated the presence of Maroteaux-Lamy syndrome. 

We explained to the patient the importance of identifying the variant in the family through genetic testing. Genetic testing was offered to both parents to avoid such complications in future pregnancies but could not be performed because of the poor affordability of parents. The patient was notified of the diagnosis and opted not to pursue treatment. No noticeable clinical changes were observed at a 6-month follow-up appointment.

## Discussion

Mucopolysaccharidosis: MPS VI (Maroteaux-Lamy syndrome) is a rare syndrome usually having an autosomal recessive inheritance pattern [4]. The main defect of this disorder lies in the deranged metabolism of GSG, DS, and CS. Usually, the patient presents with large lips, thick gingival tissue, and corneal opacities. The disease progressively causes cardiac failure and bronchopneumonia due to the accumulation of mucopolysaccharides resulting in the death of these patients [10]. The frequency of this disease ranges from 1:1.3 million to 1:1.5 million live births in the medical literature [11].

The presence of average intelligence, prominent metachromatic inclusions in leukocytes, and deficient activity of ARSB differentiate this syndrome from other mucopolysaccharidoses [8, 9]. Systemic examination in these patients reveals various musculoskeletal abnormalities such as dysostosis multiplex, genu valgum, hip dysplasia, kyphoscoliosis, joint stiffness, joint contractures, short stature, and many neurological findings absent in our case. However, respiratory findings like sinusitis, upper and lower airway obstruction, sleep apnea, and cardiac abnormalities are also present [10, 11]. Similar cardio-pulmonary insufficiency was present in our case too.

Extraoral findings include a large and prominent forehead, marked supraorbital ridges, temporal bulge, short neck, corneal opacity, enlarged skull, and a long anteroposterior dimension [9–11]. Intraorally, delayed tooth eruption, high arched palate, microdontia or malformed hypocalcified and hypoplastic teeth, thick lips, distended adenoids, and macroglossia are usually observed [8–10]. Our patient presented with similar extraoral and intraoral clinical features; however, there was no gingival hyperplasia or hypocalcified or hypoplastic teeth. In a case report, Gardner explained the presence of numerous malformed teeth with this syndrome [12]. 

Another case report described oral manifestations of Maroteaux Lammy syndrome in an 11-year-old boy with similar findings [13]. Though Gardner [12], in one of his reports, elaborated that the incidence of dental caries may be lower than average in these patients, our patient presented with a carious 23 with pulpal involvement.

Radiographic examinations are essential in the case of patients with unerupted teeth. However, a panoramic radiograph may be used in extensive lesions. CBCT is indicated for better delineation of the extent of the lesion and for establishing the lesion's relationship with adjacent anatomical structures supplemented with skull radiographs [14].

Radiographic examination of jaws reveals multiple localized radiolucent areas resembling dentigerous cyst-like follicles. Similar clearly defined radiolucent areas were detected in our patient. MacLeod SP, in their study, stated that these radiolucencies are caused by the accumulation of mucopolysaccharides in the tissues [13]. Roberts *et al.* described these osteolytic lesions as two types: the cystic type, which is destructive in nature as observed in the present case. The other type that rarely causes displacement and is limited to the crown of the unerupted tooth is the connective tissue type, mainly containing hyaluronic acid, a glycosaminoglycan that may lead to this disorder [15].

Differential diagnoses included Gorlin syndrome (basal cell nevus syndrome) with similar clinical and radiological features and bilateral paradental cyst in children. Devi P *et al.* described multiple dentigerous cysts in other syndromes such as cleidocranial dysplasia and Klippel Feil syndrome [16]. Extraoral radiographs are useful to detect osseous abnormalities and to differentiate these syndromes. Characteristic osseous changes seen in different radiographs include the absence of clavicle as seen in cleidocranial dysplasia in a posteroanterior chest x-ray; multiple wormian bones, widened sagittal sutures and/or fontanelles as seen in Cleidocranial dysplasia, calcification of falx cerebri as seen in basal cell nevus syndrome seen in an anteroposterior skull radiograph, and congenital fusion of 2 or more cervical vertebrae as seen in Klippel Feil Syndrome in an anteroposterior and lateral neck radiograph. None of these features were observed in our case.

In our patient, the presence of GAGs in the urine and deficiency of aryl sulfate B indicated the presence of mucopolysaccharide-storage disorder. The differential diagnosis of this inherited disorder includes mucolipidosis, an inherited metabolic disorder that affect the normal turnover of glycosaminoglycans and lipids in the body, and GM1 gangliosidosis, an inherited disorder that progressively destroys nerve cells (neurons) in the brain and spinal cord. These disorders can be ruled out with genetic testing of the patient [16, 17].

Management of MPS VI focuses on very early and continuous enzyme replacement therapy (ERT), which slows down the clinical course of this disorder and hematopoietic stem cell transplantation (HSCT). Literature search has shown improvement in growth rate and slow progression of cardiac parameters, and stabilization of urinary GAG levels in patients on ERT. Cystic jaw lesions are commonly treated with surgical enucleation. Several other treatment approaches, such as gene therapy and TNF-alpha antagonists, are currently being tested to be used alone or in combination with the available therapies for the maximum benefit of the patient [18].

## Conclusion

This report emphasizes the thorough evaluation of patients beyond the jaws and supports the use of CBCT to assess a lesion explicitly. MPS VI are rare inherited disorders and should be considered in the differential diagnosis of syndromes associated with multiple dentigerous cysts and other mucopolysaccharidoses. CBCT is the imaging method to evaluate the lesion and obtain relevant information for diagnosis and treatment. Genetic counseling should be encouraged in suspicious cases, and early diagnosis and multidisciplinary approach should be followed for the management of these patients to reduce morbidity.

## Acknowledgments 

### Conflict of interest

The authors declare no conflict of interest.

### Ethical approval

The case report was approved by the Institutional Review Board [Institute of Dental Studies and Technologies, Modinagar, Uttar Pradesh], and the collected data was accessible to the authors only (IDST/IERBC/CR/002/20).

### Consent to participate

The written and oral consent of the patient's mother was obtained before the documentation of the case.

### Authorship

SR, DM contributed to conceptualizing. AM, AJ contributed to the methodology. AM, DG contributed to writing the original draft. AV, AH contributed to editing the manuscript. DM, AV contributed to data curation.
